# Climatic Variation of Supercooling Point in the Linden Bug *Pyrrhocoris apterus* (Heteroptera: Pyrrhocoridae)

**DOI:** 10.3390/insects9040144

**Published:** 2018-10-19

**Authors:** Tomáš Ditrich, Václav Janda, Hana Vaněčková, David Doležel

**Affiliations:** 1 Faculty of Education, University of South Bohemia, Branisovska 31a, 37005 Ceske Budejovice, Czech Republic; vaclav.jandaa@gmail.com; 2 Biology Center, Academy of Sciences of the Czech Republic, 37005 Ceske Budejovice, Czech Republic; vanhan@seznam.cz (H.V.); dolezel@entu.cas.cz (D.D.)

**Keywords:** cold tolerance, supercooling point, overwintering, diapause

## Abstract

Cold tolerance is often one of the key components of insect fitness, but the association between climatic conditions and supercooling capacity is poorly understood. We tested 16 lines originating from geographically different populations of the linden bug *Pyrrhocoris apterus* for their cold tolerance, determined as the supercooling point (SCP). The supercooling point was generally well explained by the climatic conditions of the population’s origin, as the best predictor—winter minimum temperature—explained 85% of the average SCP variation between populations. The supercooling capacity of *P. apterus* is strongly correlated with climatic conditions, which support the usage of SCP as an appropriate metric of cold tolerance in this species.

## 1. Introduction

Population dynamics of temperate insects can be crucially affected by their survival rates during overwintering [1]. Cold tolerance, a key determinant of insect survival during winter, is thus an important research topic for insect ecologists and physiologists. This importance has increased with the recent climate changes around the world, especially in the Northern Hemisphere [2,3]. Researchers have identified many mechanisms affecting cold tolerance [4,5,6]. However, it was found to be not a single trait, but a complex set of several cold tolerance components. Cold tolerance surveys most often include determination of the supercooling point (SCP), chill coma onset (CTmin), chill coma recovery time, and lower lethal limits [7]. Nevertheless, only some of these cold tolerance metrics are ecologically relevant for particular species. For freeze-avoidant insects, prevalent in the Northern Hemisphere [8], SCP can be an appropriate metric of cold tolerance when determined correctly in cold-acclimated, overwintering stages. This especially holds for the heteropteran *Pyrrhocoris apterus* (Hemiptera: Heteroptera: Pyrrhocoridae), where SCP corresponds to lower lethal temperature [9,10,11,12].

The determinants of insects’ cold tolerance have been mainly identified under controlled laboratory conditions in model species, but less attention has been paid to supercooling capacity variation between different populations of single species. Insect populations in natural habitats might have experienced different climatic histories, which should alter individual cold tolerances according to the minimum temperatures that are usual in the populations’ areas. Hence, variation in any cold tolerance trait should indicate the relevance of the trait; if a cold tolerance component was correlated with the climatic condition of a specific population, then this cold tolerance component is ecologically relevant for that species. This was shown at a macroecological scale for many insects, interspecifically for drosophilid flies [13,14,15,16], and probably holds true for ectotherms in general [17]. In a single species, the association between climatic conditions and cold tolerance has been mainly studied in drosophilid flies and chill coma recovery time [18,19]. Small intraspecific differences in the lethal temperature (LT_50_) were found between latitudinally different populations of five drosophilid species [14]. No population differences in SCP were found for the butterfly *Atalopedes campetris* [20]. On the other hand, significant differences in SCP and lower lethal limits were found between two populations of the linden bug *Pyrrhocoris apterus* originating from localities with different climatic conditions [21]. SCP was also inversely correlated to the latitude in ten populations of the Asian corn borer *Ostrinia furnacalis* [22]. Among other arthropods, the correlations between annual mean minimum temperatures and chill coma recovery times were significant for four populations of the crustacean *Porcellio laevis* [23].

Because the results of supercooling capacity variation in populations from different climatic conditions are inconsistent, we decided to test this variation in 16 populations of the linden bug *Pyrrhocoris apterus*. The association between cold tolerance and climatic conditions of these populations would support the consideration of supercooling capacity as an important ecological trait affecting insect distribution. The winter climatic conditions include several factors, most importantly, absolute values of minimum temperatures, average temperatures, and length of the freezing period. We thus investigated which of several climatological variables can serve as the best predictor of the SCP.

## 2. Materials and Methods

### 2.1. Study Organism

The linden bug *P. apterus* is a Palearctic species with a wide distribution [24]. The physiology, especially cold tolerance, of this freeze-avoidant species has been studied for decades [16]. The SCP of this species changes throughout the season and decreases during the diapause. Although the use of SCP as only one of the cold tolerance metrics is considered problematic, especially for tropical or non-acclimated species [25], it can be used as an estimate of lower lethal temperature [9,10,11,12] in *P. apterus*.

### 2.2. Experimental Design

For purpose of this study, we used 16 populations from geographically different locations, established and reared in the Biology Center, Academy of Sciences of the Czech Republic (see Table 1 and [26] for detailed information of original populations and sample sizes). All field lines were kept in constant light conditions at 24–26 °C in 250 mL glass jars on dry linden seed (*Tilia cordata*), with wet cotton as a source of water. Because the diapause is needed for reaching maximal cold tolerance [10], the experimental generation was removed from these lines as third instar larvae and kept at 20 °C in short-day conditions (SD, 12:12). Two-week-old adults were exposed to SD photoperiod combined with following fluctuating thermal regimes: two weeks of 20 °C/10 °C, followed by two weeks of 15 °C/5 °C, and additional two weeks of 10 °C/0 °C. After these six weeks of thermoperiodic acclimation, the bugs were kept for an additional 3–4 weeks at 0 °C in constant dark conditions. This protocol has been used earlier for diapause induction in *P. apterus* (TD, unpublished). The SCP was determined for every individual according to the protocol described in [9]: the individuals were dried using cotton tissue and inserted into 1.5-mL Eppendorf microtubes, fitted with thermocouples (type K) attached to TC-08 Picolog Thermocouple Data Loggers (Pico Technology, St Neots, Cambridgeshire, UK). The bugs were immobilized with cotton wool. The microtubes were then placed into aluminum blocks cooled by an ethanol bath pre-cooled to 0 °C. They were then cooled to −30 °C at a rate of 0.2 °C/min and all exotherms indicating freezing of body fluids were recorded. All handling prior to the SCP measurement was done on the crushed ice to prevent warming of the specimens. After freezing, every individual was sexed and fresh mass (FM) was measured. The SCP was determined in winter 2016 for all populations except three, which were collected during 2016 and tested in winter 2017.

### 2.3. Climatic Data

For every location, climatic data from January and February (which are generally the coldest months) for 2010–2015 were used. When available, official meteorological institutes were used as data sources. Nevertheless, these data were not available for some localities. In that case, the historical data for 2010–2015 were accessed from the Weather Underground meteorological portal (see Table 1 for details). Particular climatic data used as explanatory variables in this study were: average winter temperature (Tav); minimum winter temperature (Tmin); number of freezing days (T < 0; defined as days with minimum temperature below 0 °C); and number of −5 °C days (T < −5; defined as days with minimum temperature below −5 °C). All these data were based on January and February of 2010–2015.

### 2.4. Statistical Analysis

At first, a general linear model (GLM) was computed with SCP as dependent variable; sex and population (sex nested in population, population as factor with random effect) as categorical variables; and fresh mass as a continuous variable. The association between climatological data (Tav; Tmin; T < 0; T < −5) and SCP was assessed as the linear regression of the mean SCP (all individuals from one population pooled) and particular climatological variable. Moreover, a PCA of climatological variables was performed and the SCP was correlated with the localities coordinates on the first axis of PCA. The statistical tests were performed using Statistica 13 (Tibco Software, Palo Alto, CA, USA).

## 3. Results

**Figure 1:**
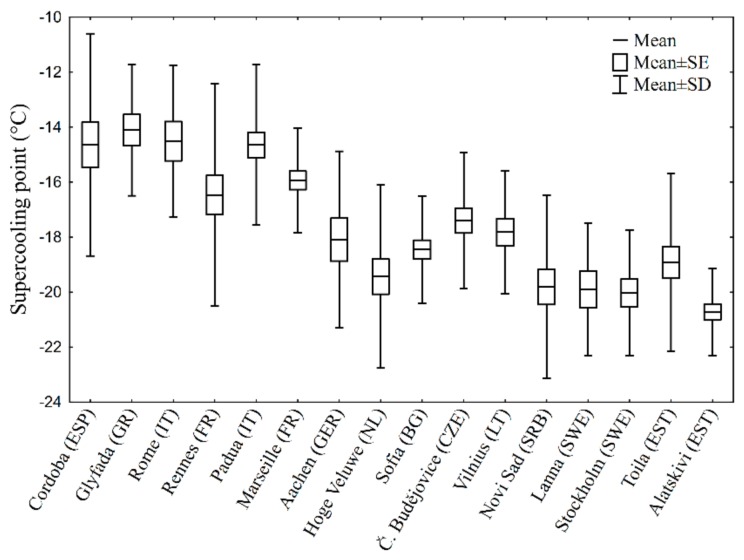
Distribution of supercooling points (SCPs) among populations (the localities are ordered by decreasing minimal winter temperature). See Table 1 for sample sizes.

The mean population SCP was highest in the southern populations (Greece, Italy, and Spain), whereas the lowest values were reached in northern populations (Estonia and Sweden) (Figure 1). The GLM revealed a significant effect of population (F_15, 378_ = 21.31; *p* < 10^−7^) and fresh mass (F_1, 378_ = 6.71; *p* < 10^−3^), but non-significant effect of sex (F_16, 378_ = 0.68; *p* = 0.82).

**Figure 2:**
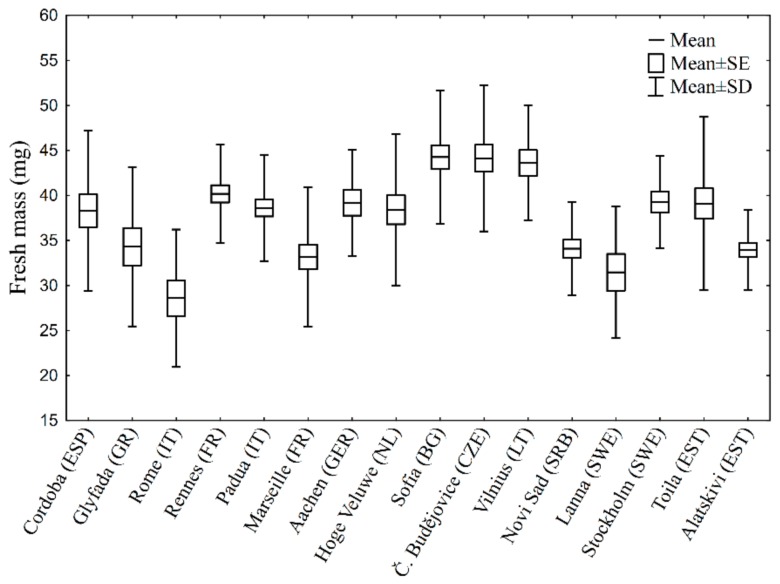
Variation in fresh mass among populations (the localities are ordered by decreasing minimal winter temperature). See Table 1 for sample sizes.

The effect of fresh mass was tested further with linear regression. The overall association between fresh mass and SCP is significant (*p* = 0.03; r = −0.1). According to the regression equation, every 10 mg of fresh mass added results in a decreased SCP by 0.45 °C. Because the SCP differences by 0.5 °C can be considered negligible [9], the relevance of this correlation seems unimportant. Moreover, when the correlation was investigated in separate populations, it was significant only in two out of 16 populations (Rennes and Toila). The fresh mass variation differs among populations, but the pattern of fresh mass variation lacks clear dependence on climatological factors (Figure 2). Hence, the effect of fresh mass was not considered further in separate analyses.

**Figure 3:**
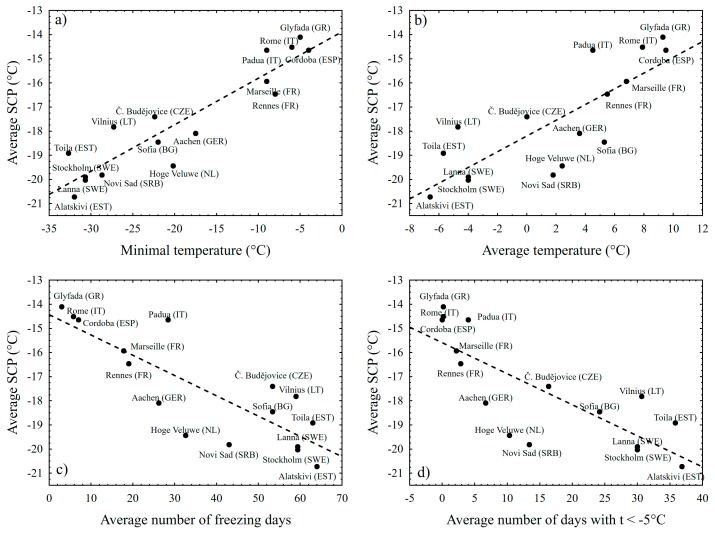
Average population SCP as a linear function of climatic conditions (January–February during 2010–2015). All investigated climatic factors are significantly correlated with the mean population SCP, but the extent of explained variability slightly differs: (**a**) strong correlation with minimum temperature explained 85% of SCP variability; (**b**) the winter average temperature explained 64% of SCP variability; (**c**) the number of freezing days explained 69% of SCP variability; and (**d**) number of days with minimum temperature below −5 °C explained 63% of SCP variability.

The association between climatic factors and average population SCP was significant in all cases (Figure 3a–d). The strongest correlation (r = 0.92, *p* < 10^−7^) was found between the average SCP and the minimum winter temperature (Figure 3a). The average temperature was also correlated with average population SCP significantly, but the association was weaker (r = 0.8; *p* < 10^−3^) (Figure 3b). In addition, the number of cold days correlates with average population SCP; number of freezing days with r = −0.83 and *p* < 10^−4^ (Figure 3c); but the number of days with the minimum temperature below −5 °C was slightly weaker (r = −0.79; *p* < 10^−3^) (Figure 3d). South European populations—in Greece (Glyfada), Italy (Padua and Rome), Spain (Cordoba), and Southern France (Marseille)—had not entered the diapause upon SD as eggs were found among the experimental generation. It is not clear if all or only some individuals of South European populations remained reproductive in conditions inducing diapause in Central and North European populations. The correlation between Tmin and SCP was significant (r = 0.77, *p* = 0.006) even after the exclusion of the Southern (Greek, Italian, Spanish, and Southern French) populations.

**Figure 4:**
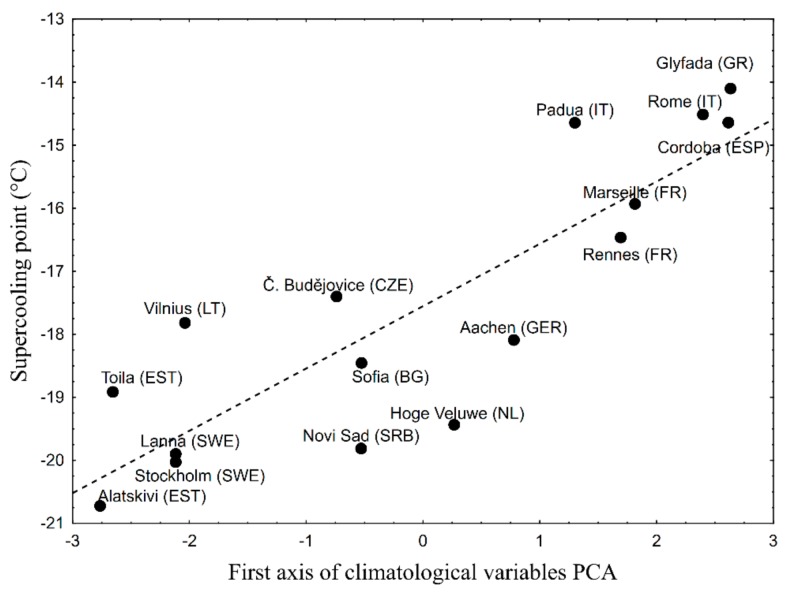
Average population SCP as a linear function of first axis of climatological variables PCA. The correlation is significant and the first PCA axis explained 74% of SCP variability.

The PCA of climatological variables yielded into first axis, explaining 95% of the variability. The correlation between this first PCA axis and SCP was significant (r = 0.86; *p* < 10^−4^) and the first PCA axis explained 74% of SCP variability (Figure 4).

## 4. Discussion

The association between climatic conditions and the average population supercooling capacity is strong and obvious for all climatic factors tested in this study. Given that each data point is actually a population mean makes this relationship even stronger. Out of these climatic factors, the value of minimal recorded temperature seems to be the best variable explaining supercooling capacity. This is in accordance with a general consideration of extreme temperatures as being more important than average temperatures [27,28]. However, the relation between minimum temperature and SCP is not straightforward; the SCP is usually 10 °C lower than the minimum temperature (Figure 3a). This difference could be explained in terms of imperfect temperature record; the minimum temperature used in this study is a record from January and February for 2010–2015; the populations could have had experienced lower temperatures in history.

### Limitation of this Study

The populations used in this study differ not only in the climatic conditions at their original location, but importantly also in their ability to enter the reproductive diapause. Whereas *P. apterus* from Central and Northern Europe enter diapause upon exposure to short photoperiods, populations from South Europe are often photoperiod insensitive [29,30]. Indeed, the South European populations with generally high SCPs—in Greece (Glyfada), Italy (Padua and Rome), Spain (Cordoba), and Southern France (Marseille)—had not entered the diapause upon SD conditions, as eggs were found among the experimental generation. It is thus clear that not all, if any, individuals entered diapause in conditions inducing diapause in Central and North European populations. The diapause is a prerequisite for maximization of the cold tolerance in this species (SCP decreases by ~10 °C during diapause termination, compared to reproduction phase), other heteropteran *Graphosoma lineatum* and other insects [10,31,32] The absence of diapause in some populations could be the main factor responsible for relatively low supercooling capacity. It is unclear whether individuals from these Mediterranean populations are not generally able to enter diapause or if the diapause was induced later at low temperatures during the acclimation protocol. Nevertheless, the correlation between Tmin and SCP was significant (r = 0.77, *p* = 0.006) even after the exclusion of the Southern (Greek, Italian, Spanish, and Southern French) populations. Therefore, populations where SD induces diapause still differs in SCP, which correlates with the climatic conditions of their origin. However, we cannot exclude the possibility that these photoperiodically-sensitive populations would have even lower SCPs if they were reared under different diapause—inducing conditions. In principle, every single population might have its own optimal temperature and photoperiodic conditions inducing its maximal cold tolerance. It is theoretically possible that maximal cold tolerance was not reached in most populations and that theoretical maximal supercooling capacity does not differ. These concerns are ecologically meaningful, but extremely demanding from a practical standpoint. Here, the presented study relied on a simple protocol when all populations were reared and exposed to the same conditions. The results should be interpreted with the limitation that every population (or even every individual) might have its own specific acclimation protocol, maximizing its cold tolerance (supercooling capacity).

## 5. Conclusions

In conclusion, the strong association between winter climatic conditions and SCP in geographically different populations of *P. apterus* supports the view of insect diapause as an important determinant of cold tolerance [26], and suggests the possibility of cold tolerance as one of the key life-history traits, likely affecting population performance and species distribution. The results also support the relevance of SCP as an indicator of cold tolerance in the linden bug, *P. apterus*.

## Figures and Tables

**Table 1:** A list of populations origin and climatic data sources. All populations, with exception of the Vilnius population, were kept in the laboratory for at least three generations.

Population	GPS	Generation	Sampling Date	Climatic Data Source	*n*
Aachen (GER)	50°49′56.8″ N	F7	06/2014	The Royal Netherlands Meteorological Institute	17
	06°03′01.6″ E				
Alatskivi (EST)	58°35′31.7″ N	F31	06/2010	The Estonian Environment Agency	32
	27°07′29.8″ E				
Cordoba (ESP)	37°53′21.5″ N	F17	08/2012	Weather Underground	24
	04°47′51.7″ W				
Č. Budějovice (CZE)	48°59′37.3″ N	F3	08/2014	Czech Hydrometeorological Institute	30
	14°32′30.1″ E				
Glyfada (GR)	37°52′46.1″ N	F33	08/2009	Helenic National Meteorological Service	18
	23°46′06.2″ E				
Hoge Veluwe (NL)	52°04′57.7″ N	F19	06/2012	The Royal Netherlands Meteorological Institute	27
	05°50′00.2″ E				
Lanna (SWE)	59°12′00.0″ N	F36	04/2010	Weather Underground	13
	18°09′00.0″ E				
Marseille (FR)	43°17′35.9″ N	F58	06/2004	Weather Underground	32
	05°21′35.6″ E				
Novi Sad (SRB)	45°15′41.4″ N	F30	06/2011	Republic Hydrometeorological Service of Serbia	27
	19°51′19.4″ E				
Padua (IT)	45°24′34.8″ N	F4	07/2016	Weather Underground	40
	11°53′53.3″ E				
Rennes (FR)	48°06′51.8″ N	F3	05/2016	Weather Underground	32
	1°38′08.3″ W				
Rome (IT)	41°55′52.8″ N	F4	06/2016	Weather Underground	15
	12°28′54.4″ E				
Sofia (BG)	42°42′00.0″ N	F27	07/2011	Weather Underground	32
	23°19′12.0″ E				
Stockholm (SWE)	59°12′00.0″ N	F36	04/2010	Weather Underground	20
	18°09′00.0″ E				
Toila (EST)	59°24′57.6″ N	F33	06/2010	The Estonian Environment Agency	32
	27°31′02.3″ E				
Vilnius (LT)	54°41′41.9″ N	F0	10/2015	Lithuanian Hydrometeorological Service	20
	25°15′34.8″ E				

## References

[1] Leather S.R., Walters K.F.A., Bale J.S. (1993). The Ecology of Insect Overwintering.

[2] Robinet C., Roques A. (2010). Direct impacts of recent climate warming on insect populations. Integr. Zool..

[3] Bale J.S., Hayward S.A.L. (2010). Insect overwintering in a changing climate. J. Exp. Biol..

[4] Clark M.S., Worland M.R. (2008). How insects survive the cold: Molecular mechanisms—A review. J. Comp. Physiol. B.

[5] Storey K.B., Storey J.M. (2012). Insect cold hardiness: Metabolic, gene, and protein adaptation. Can. J. Zool..

[6] Denlinger D.L., Lee R.E. (2010). Low Temperature Biology of Insects.

[7] Sinclair B.J., Alvarado L.E.C., Ferguson L.V. (2015). An invitation to measure insect cold tolerance: Methods, approaches, and workflow. J. Therm. Biol..

[8] Sinclair B.J., Chown S.L. (2005). Climatic variability and hemispheric differences in insect cold tolerance: Support from southern Africa. Funct. Ecol..

[9] Ditrich T. (2018). Supercooling point is an individually fixed metric of cold tolerance in *Pyrrhocoris apterus*. J. Therm. Biol..

[10] Košťál V., Šimek P. (2000). Overwintering strategy in *Pyrrhocoris apterus* (Heteroptera): The relations between life-cycle, chill tolerance and physiological adjustments. J. Insect Physiol..

[11] Nedvěd O., Hodková M., Brunnhofer V., Hodek I. (1995). Simultaneous measurement of low-temperature survival and supercooling in a sample of insects. Cryoletters.

[12] Hodková M., Hodek I. (1997). Temperature regulation of supercooling and gut nucleation in relation to diapause of *Pyrrhocoris apterus* (Heteroptera). Cryobiology.

[13] Addo-Bediako A., Chown S.L., Gaston K.J. (2000). Thermal tolerance, climatic variability and latitude. Proc. R. Soc. Lond. B Biol. Sci..

[14] Kimura M.T. (2004). Cold and heat tolerance of drosophilid flies with reference to their latitudinal distributions. Oecologia.

[15] Kellermann V., Loeschcke V., Hoffmann A.A., Kristensen T.N., Flojgaard C., David J.R., Svenning J.C., Overgaard J. (2012). Phylogenetic constraints in key functional traits behind species’ climate niches: Patterns of desiccation and cold resistance across 95 drosophila species. Evolution.

[16] Andersen J.L., Manenti T., Sørensen J.G., MacMillan H.A., Loeschcke V., Overgaard J., Woods A. (2015). How to assess *Drosophila* cold tolerance: Chill coma temperature and lower lethal temperature are the best predictors of cold distribution limits. Funct. Ecol..

[17] Sunday J.M., Bates A.E., Dulvy N.K. (2011). Global analysis of thermal tolerance and latitude in ectotherms. Proc. R. Soc. Lond. B Biol. Sci..

[18] David J.R., Gibert P., Moreteau B., Gilchrist G.W., Huey R.B. (2003). The fly that came in from the cold: Geographic variation of recovery time from low-temperature exposure in *Drosophila subobscura*. Funct. Ecol..

[19] Sinclair B.J., Williams C.M., Terblanche J.S. (2012). Variation in thermal performance among insect populations. Physiol. Biochem. Zool..

[20] Crozier L. (2003). Winter warming facilitates range expansion: Cold tolerance of the butterfly *Atalopedes campestris*. Oecologia.

[21] Kalushkov P., Nedvěd O. (2000). Cold hardiness of *Pyrrhocoris apterus* (Heteroptera: Pyrrhocoridae) from central and southern europe. Eur. J. Entomol..

[22] Xie H.C., Li D.S., Zhang H.G., Mason C.E., Wang Z.Y., Lu X., Cai W.Z., He K.L. (2015). Seasonal and geographical variation in diapause and cold hardiness of the asian corn borer, *Ostrinia furnacalis*. Insect Sci..

[23] Castaneda L.E., Lardies M.A., Bozinovic F. (2005). Interpopulational variation in recovery time from chill coma along a geographic gradient: A study in the common woodlouse, *Porcellio laevis*. J. Insect Physiol..

[24] Socha R. (1993). *Pyrrhocoris apterus* (Heteroptera)—An experimental model species: A review. Eur. J. Entomol..

[25] Renault D., Salin C., Vannier G., Vernon P. (2002). Survival at low temperatures in insects: What is the ecological significance of the supercooling point?. Cryoletters.

[26] Pivarciova L., Vaněčková H., Provaznik J., Wu B.C.H., Pivarci M., Pecková O., Bazalová O., Cada S., Kment P., Kotwica-Rolinska J. (2016). Unexpected geographic variability of the free running period in the linden bug *Pyrrhocoris apterus*. J. Biol. Rhythm..

[27] Parmesan C., Root T.L., Willig M.R. (2000). Impacts of extreme weather and climate on terrestrial biota. Bull. Am. Meteorol. Soc..

[28] Bale J.S. (1996). Insect cold hardiness: A matter of life and death. Eur. J. Entomol..

[29] Numata H., Saulich A.H., Volkovich T.A. (1993). Photoperiodic responses of the linden bug, *Pyrrhocoris apterus*, under conditions of constant-temperature and under thermoperiodic conditions. Zool. Sci..

[30] Saunders D.S. (1983). A diapause induction termination asymmetry in the photoperiodic responses of the linden bug, *Pyrrhocoris apterus* and an effect of near-critical photoperiods on development. J. Insect Physiol..

[31] Šlachta M., Vambera J., Zahradníčková H., Košťál V. (2002). Entering diapause is a prerequisite for successful cold-acclimation in adult *Graphosoma lineatum* (Heteroptera: Pentatomidae). J. Insect Physiol..

[32] Denlinger D.L., Lee R.E., Denlinger D.L. (1991). Relationship between cold hardiness and diapause. Insects at Low Temperature.

